# Integrin α9 gene promoter is hypermethylated and downregulated in nasopharyngeal carcinoma

**DOI:** 10.18632/oncotarget.5154

**Published:** 2015-09-08

**Authors:** Imran Nawaz, Li-Fu Hu, Zi-Ming Du, Khalid Moumad, Ilya Ignatyev, Tatiana V. Pavlova, Vladimir Kashuba, Malin Almgren, Eugene R. Zabarovsky, Ingemar Ernberg

**Affiliations:** ^1^ Department of Microbiology, Tumor and Cell Biology, Karolinska Institutet, Stockholm, Sweden; ^2^ Department of Microbiology, Faculty of Life Sciences, University of Balochistan, Quetta, Pakistan; ^3^ State Key Laboratory of Oncology in South China, and Department of Pathology, Sun Yat-Sen University Cancer Center, Guangzhou, P.R. China; ^4^ Department of Molecular Genetic Epidemiology, German Cancer Research Center (DKFZ), Heidelberg, Germany; ^5^ Oncovirology Laboratory, Institut Pasteur du Maroc, Casablanca, Morocco; ^6^ Department Clinical Neuroscience, Karolinska Institutet, Stockholm, Sweden; ^7^ Centre for Molecular Medicine, Stockholm, Sweden; ^8^ Department of Clinical & Experimental Medicine, Division of Cell Biology, Linköping University, Linköping, Sweden

**Keywords:** nasopharyngeal carcinoma, ITGA9, DNA methylation, notI microarrays, epigenetics

## Abstract

Epigenetic silencing of tumor suppressor genes (TSGs) by promoter methylation can be an early event in the multi-step process of carcinogenesis. Human chromosome 3 contains clusters of TSGs involved in many cancer types including nasopharyngeal carcinoma (NPC), the most common cancer in Southern China. Among ten candidate TSGs identified in chromosome 3 using NotI microarray, ITGA9 and WNT7A could be validated. 5′-aza-2′ deoxycytidine treatment restored the expression of ITGA9 and WNT7A in two NPC cell lines. Immunostaining showed strong expression of these genes in the membrane and cytoplasm of adjacent control nasopharyngeal epithelium cells, while they were weakly expressed in NPC tumor cells. The ITGA9 promoter showed marked differentially methylation between tumor and control tissue, whereas no differentially methylation could be detected for the WNT7A promoter. The expression level of ITGA9 in NPC tumors was downregulated 4.9-fold, compared to the expression in control. ITGA9 methylation was detected by methylation specific PCR (MSP) in 56% of EBV positive NPC- cases with 100% specificity. Taken together, this suggests that ITGA9 might be a TSG in NPC that is involved in tumor cell biology. The possibility of using ITGA9 methylation as a marker for early detection of NPC should further be explored.

## INTRODUCTION

Nasopharyngeal carcinoma (NPC) is a malignant tumor that arises in the surface epithelium of the posterior wall of the nasopharynx [1]. Its clinical presentation, epidemiology, and histopathology are different from typical squamous cell carcinomas of the head and neck [2]. NPC has a marked ethnic and geographic distribution. Specifically, its most common form, WHO type III is highly prevalent in Southern China, Southeast Asia, North Africa and Greenland, and shows a close to 100% prevalence of Epstein-Barr virus (EBV) in the tumor cells [3–7]. Regardless of the geographic distribution, NPC is suggested to result from the contribution and interplay of at least three distinctly different factors including environmental, genetic as well as EBV infection [8].

NPC patients in stages I and II of the disease have a significantly longer overall survival compared with those in stages III and IV. The five-year survival rate for stage III or IV patients is only around 40 to 50%, while it can be as high as 95% for stage I or II patients [9–11]. Because of non-specific local symptoms and the inconvenience of full clinical examination of the nasopharynx, approximately 70% of NPC patients are only diagnosed when the tumor has reached an advanced stage with a poor prognosis [3, 10, 12]. Thus, early diagnosis of NPC is essential to achieve satisfactory treatment outcome. Finding biomarkers for detection of NPC at an early stage and monitoring recurrent tumor would contribute significantly to improve survival and guide the choice of subsequent therapy, particularly in the high incidence regions.

Changes in DNA methylation play an important role in both normal development and differentiation as well as cancer and several other diseases [13]. Alterations in DNA methylation with resulting aberrant activation of oncogenes or inactivation of certain TSGs is a key event in cancer development [14, 15]. It has recently been suggested that cancer can be initiated by an epigenetic process before any mutations [16]. These TSGs were shown to be involved in fundamental pathways, such as apoptosis, DNA damage repair, tumor invasion and metastasis, cell cycle control and intracellular adhesion [17]. Silencing of potential TSGs by aberrant methylation of CpG-rich promoter regions seems to be a key mechanism in NPC carcinogenesis [18–20]. It might be useful as a powerful means for the early diagnosis of NPC [21, 22]. The establishment of methylation based techniques for the early diagnosis of cancer will increase the chances for treatment and improve the survival rate.

Several approaches that allow high-throughput analyses of multiple CpG-rich regions in gene promoters were recently developed, including methylation specific oligonucleotide microarrays, restriction landmark genomic scanning, and differential methylation hybridization [23–27]. Loss of heterozygosity (LOH) and cytogenetic studies earlier showed that frequent changes in chromosome 3 are common in many tumors, harboring several TSGs [28]. A NotI microarray developed by our group provides another possibility to search for methylation related genes in chromosome 3 [29]. The main idea of the approach is that the NotI restriction enzyme cuts only unmethylated CpG pairs inside the recognition sequence of the enzyme (5′-GCGGCCGC-3′). Only a small fraction (0.1–0.5%) of the human genome contains NotI restriction sites, often located to CpG-islands [30]. Thus by using NMAs (glass microarrays with attached NotI-sequence tagged DNA fragments) we could compare the methylation status of control and malignant cells at the genomic level.

In this study, using chromosome 3 NMA, 188 putative TSGs were examined in NPC tumors and cell lines, and by subsequent analysis ITGA9- and WNT7A- control regions were shown to be hypermethylated and downregulated.

## RESULTS

### Screening for potentially methylated genes in NPC using NotI microarrays

NotI microarrays were performed to screen the potentially methylated genes in chromosome 3 in NPC. Genomic DNA from three NPC tumor biopsies (T9, T10 and T18), two control nasopharyngeal epithelial tissues (N1 and N2), three NPC cell lines (CNE1, TWO3 and C666–1) and one control nasopharyngeal epithelium derived cell line NP69 were used for this screening. Ten genes (ALDH1L1, BCL6, EPHB3, FGD5, FGF12, ITGA9, NUDT16P, RBSP3, WNT7A and ZIC4) were identified showing reduced signal in NPC samples as compared to control samples suggesting methylation or deletion of these genes in NPC (Table 1). To investigate the methylation status of these genes and possible correlation with gene downregulation, methylation specific PCR (MSP) and quantitative real time PCR (Q-PCR) were then performed. On the basis of the correlations found between the MSP and Q-PCR results two genes i.e. ITGA9 and WNT7A were selected for further validation and investigation.

**Table 1:** Screening for potentially methylated genes in NPC using NotI microarrays

Spot No	Gene	Loci	T10/N1	T18/N2	T9/NP69	CNE1/NP69	TWO3/NP69	C666–1/NP69
NotI0008	VHL	3p26–p25						
NotI0019	WNT7A	3p25						
NotI0021	FGD5	3p24.3						
NotI0026	PLCL2	3p24.3						
NotI0038	CLASP2	3p23						
NotI0039	LBA1	3p22.3						
NotI0040	ITGA9	3p21.3						
NotI0041	RBSP3	3p21.33						
NotI0061	SEMA3F	3p21.3						
NotI0074	BHLHB2	3p26						
NotI0085	FOXP1	3p14.2						
NotI0086	PDZRN3	3p12.3						
NotI0091	MINA	3q11.2						
NotI0100	LRRC58	3q13.33						
NotI0105	ALDL1L1	3q21.2						
NotI0110	GATA2	3q21.3						
NotI0122	NUDT16P	3q21.3						
NotI0129	SOX14	3q22						
NotI0137	PAQR9	3q23						
NotI0139	ZIC4	3q24						
NotI0140	SIAH2	3q25						
NotI0143	GMPS	3q24						
NotI0149	SOX2	3q26.3						
NotI0159	EPHB3	3q21						
NotI0163	BCL6	3q27						
NotI0166	FGF12	3q28						
NotI0175	PIGX	3q29						
NotI0176	PCBP4	3p21						
NotI0181	SIMP	3p24.1						
NotI0182	SLC22A13	3p21.3						
								
Amplification		Normal		Heterozygous Del/Met (0.85)		Homozygous Del/Met (0.35)		Non-informative

NotI microarrays were performed on genomic DNA from three NPC biopsies (T), two control nasopharyngeal epithelial tissues (N), three NPC cell lines and one cell line derived from control nasopharyngeal epithelium. A representative selection of genes is shown. Key to the different shades used in the cells is given at the bottom of the table.

### Demethylation and restoration ITGA9 and WNT7A expression in NPC cell lines by demethylating reagent 5′-aza-C

**Figure 1:**
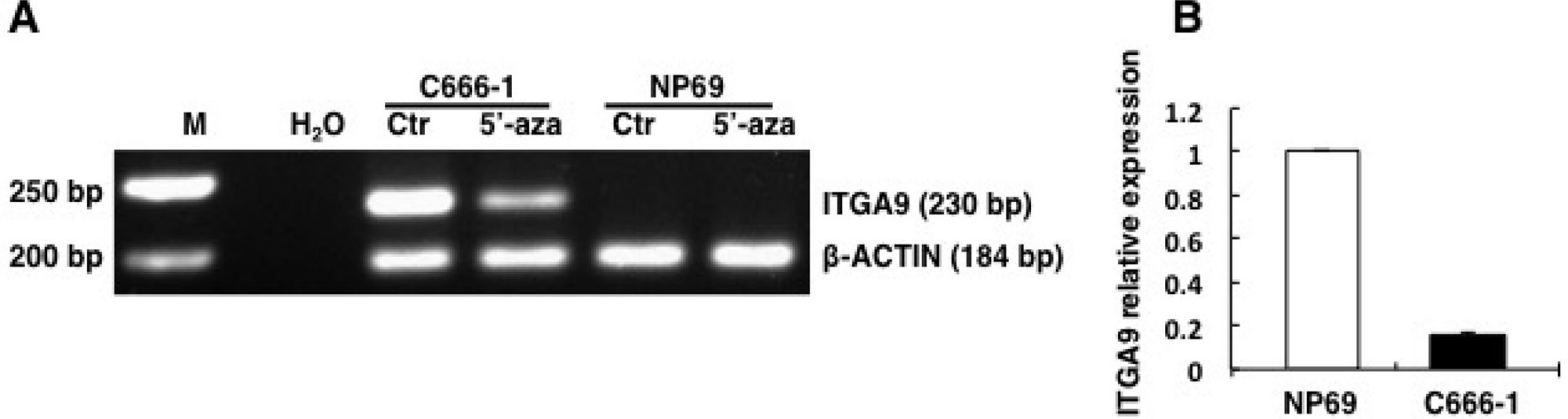
ITGA9 promoter is demethylated by demethylation reagent 5′-aza-C in NPC C666–1 cell line where its expression is also downregulated **A.** Methylation status of ITGA9 analyzed in control nasopharyngeal epithelium derived cell line NP69 and NPC C666-1 cell line treated with 5′-aza-C by MSP. **B.** mRNA expression status of ITGA9 analyzed in control nasopharyngeal epithelium derived cell line NP69 and NPC C666-1 cell line by Q-PCR.

**Figure 2:**
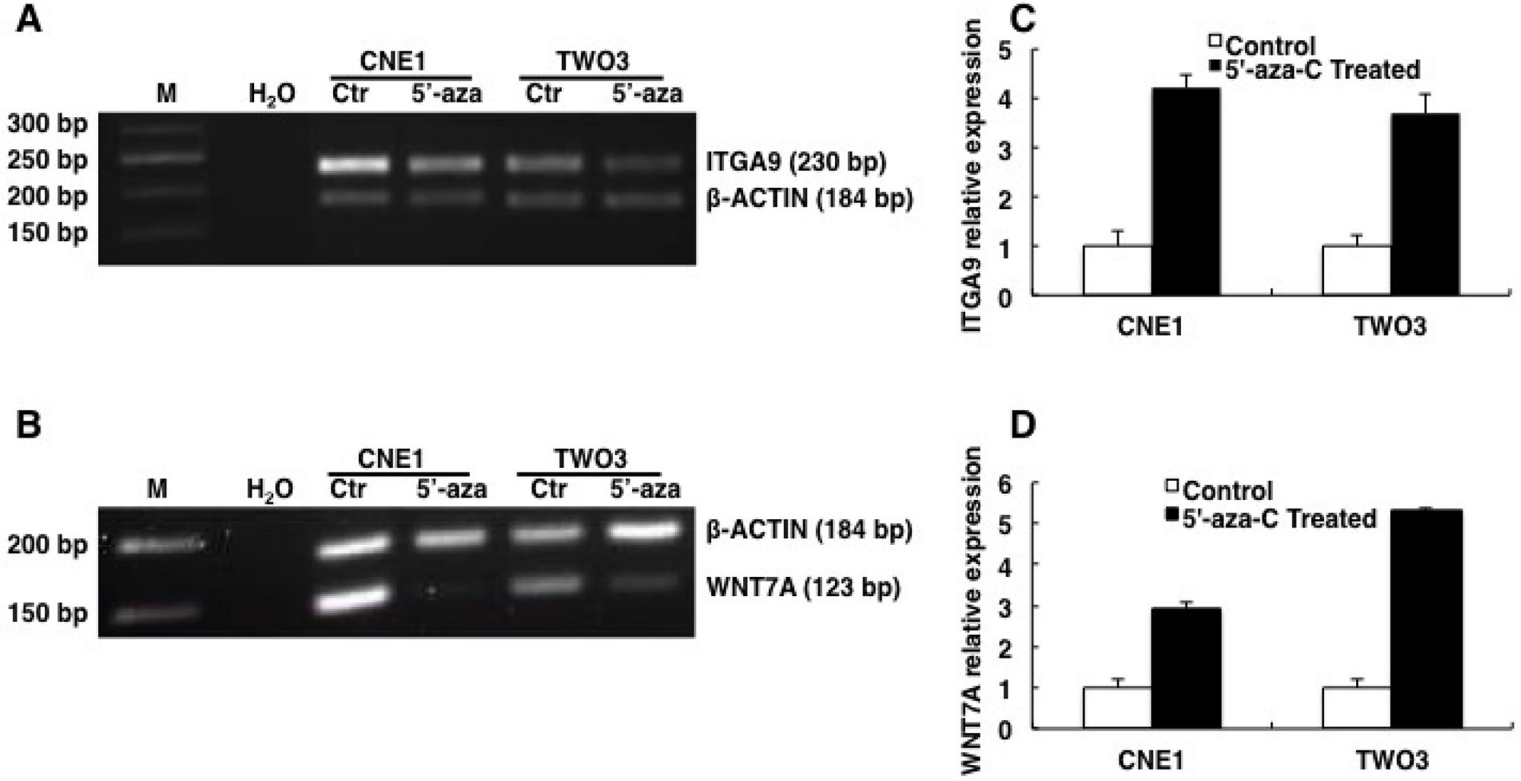
ITGA9 and WNT7A are restored by demethylation reagent 5′-aza-C in NPC cell lines Methylation status of ITGA9 **A.** and WNT7A **B.** analyzed in NPC CNE1 and TWO3 cell lines treated with 5′-aza-C by MSP. mRNA expression status of ITGA9 **C.** and WNT7A **D.** analyzed in 5′-aza-C treated and untreated NPC cell lines CNE1 and TWO3 by Q-PCR.

To investigate whether demethylating reagent 5′-aza-C could demethylate ITGA9 and WNT7A promoters, NPC C666–1, CNE1, TWO3 cell lines and control nasopharyngeal epithelium derived cell line NP69 were treated with 10 μM of the 5′-aza-C for 96 h, and then MSP was performed to detect methylation status of ITGA9 and WNT7A. It was observed that the CpG-rich regions in the promoter of ITGA9 and WNT7A were demethylated in NPC cell lines after 5′-aza-C treatment by MSP, compared with untreated cells (Figure 1A & 2A). A comparison of ITGA9 expression in NP69 and C666–1 cell lines showed that the expression level of ITGA9 mRNA was downregulated in NPC C666–1 (6.4 ± 0.1 fold) as compared to control cell line (Figure 1B).

Furthermore Q-PCR was then performed to detect ITGA9 and WNT7A mRNA expression (Figure 2C & 2D) in 5′-aza-C treated and untreated NPC CNE1 and TWO3 cell lines. The expression level of ITGA9 mRNA was upregulated in NPC CNE1 and TWO3 after 5′-aza-C treatment (4.2 ± 0.3 fold; 3.8 ± 0.3 fold respectively) as compared to untreated controls. The expression level of WNT7A was also upregulated in NPC CNE1 and TWO3 after 5′-aza-C treatment (3.1 ± 0.2 fold; 5.6 ± 0.1 fold respectively) as compared to untreated controls. Thus demethylating reagent 5′-aza-C could restore the expression of ITGA9 and WNT7A in NPC cell lines. This suggests that the downregulation of ITGA9 and WNT7A in NPC cell lines could be due to hypermethylation of promoters of these genes and not because of deletion of these genes in these cell lines.

### Downregulation of ITGA9 and WNT7A were found in NPC samples by immunostaining

**Figure 3:**
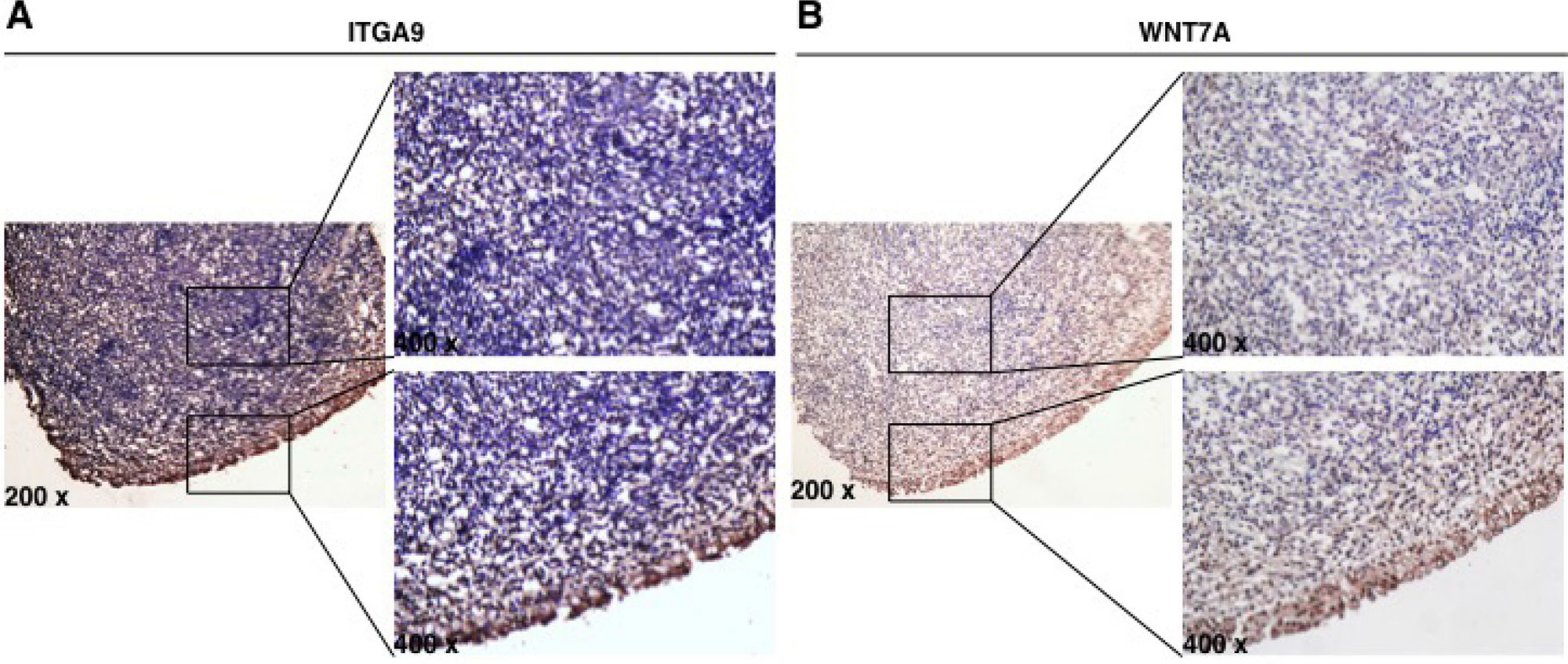
ITGA9 and WNT7A are downregulated in NPC clinical samples Expression of ITGA9 **A.** and WNT7A **B.** evaluated in NPC tumor cells and adjacent control nasopharyngeal epithelium by immunostaining.

Immunostaining was performed to evaluate the expression of ITGA9 and WNT7A proteins in three cases of NPC tumor cells and adjacent control nasopharyngeal epithelium. Strong expression of ITGA9 (Figure 3A) and WNT7A (Figure 3B) was observed in membrane and cytoplasm of adjacent control nasopharyngeal epithelium cells, while weak or no expression of ITGA9 and WNT7A was observed in NPC tumor cells. This suggests that ITGA9 and WNT7A are downregulated in NPC tumors as compared to normal cells.

### Hypermethylation of ITGA9 and WNT7A in NPC

**Figure 4:**
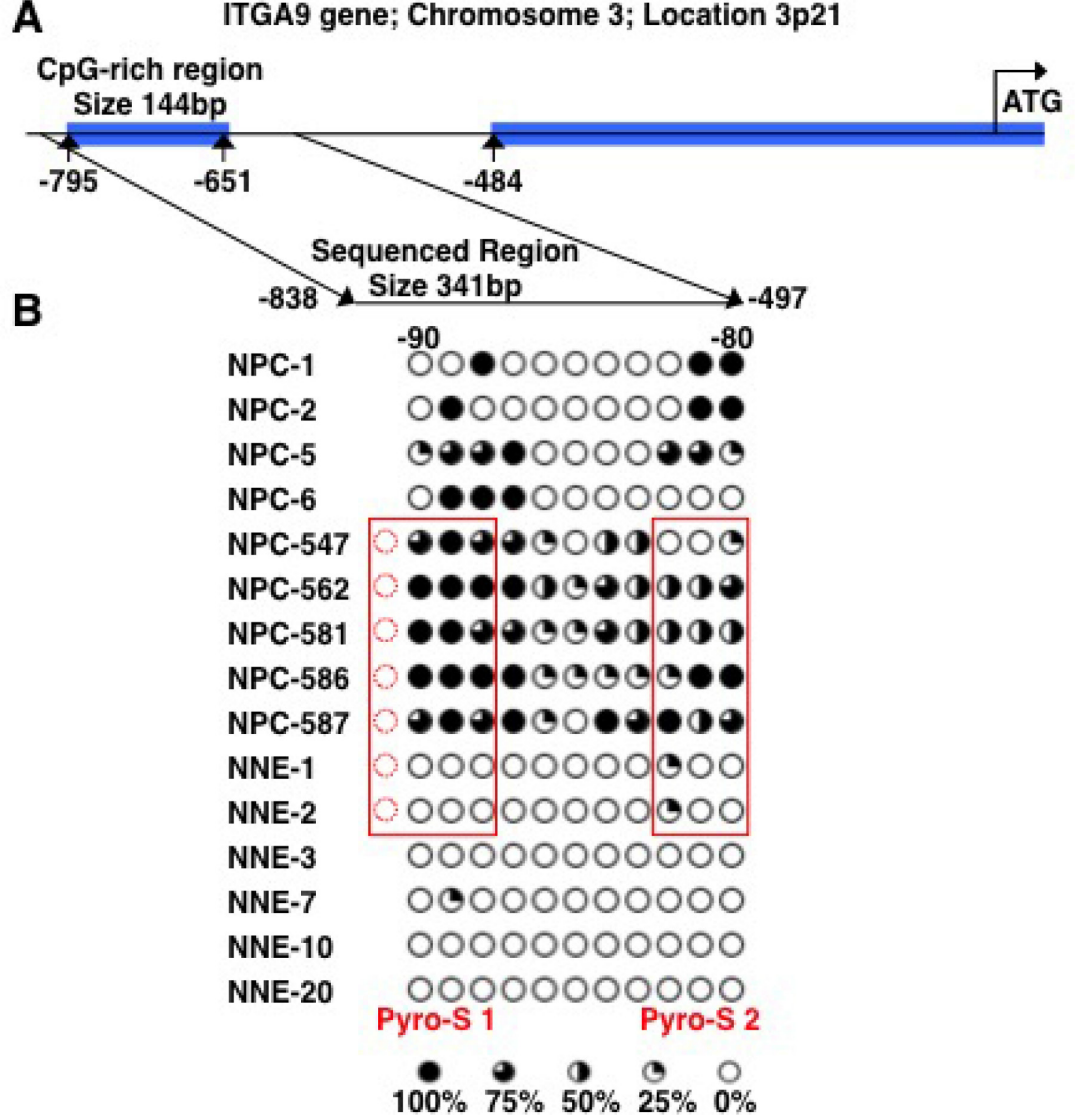
ITGA9 is differentially hypermethylated in NPC clinical samples **A.** Schematic representation of ITGA9 gene and location of the CpG-rich regions in its promoter. **B.** Eleven CpG sites in the ITGA9 promoter analyzed by sequencing the clones derived from bisulfite converted DNA. Five to ten clones were sequenced for each sample. Open and filled circles represent unmethylated and methylated CpG sites, respectively. Circles are filled according to the percentage of clones with methylation of the CpG sites. The samples marked with red boxes were used also for the sequence analyses of bisulfite converted DNA by pyrosequencing assays (see figure 6).

**Figure 5:**
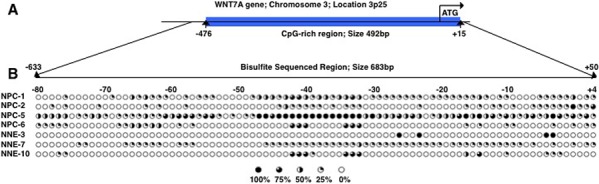
WNT7A is partially methylated in NPC clinical samples **A.** Schematic representation of WNT7A gene and the location of a CpG-rich region in its promoter. **B.** Eighty-four CpG sites in WNT7A promoter were analyzed by sequencing the clones derived from bisulfite converted DNA. Five to ten clones were sequenced for each sample. Open and filled circles represented unmethylated and methylated CpG sites, respectively. Circles are filled according to the percentage of clones with methylation of the CpG sites.

The methylation status of ITGA9 and WNT7A promoters in NPC tumor biopsy samples was investigated by sequencing of clones after bisulfite conversion. A CpG-rich region in the promoter of ITGA9 gene was investigated (Figure 4A). This region containing 11 CpG sites and was sequenced in nine NPC biopsies (NPC-1, NPC-2, NPC-5, NPC-6, NPC-547, NPC-562, NPC-581, NPC-586 and NPC-587) and six control nasopharyngeal epithelia biopsy (NNE-1, NNE-2, NNE-3, NNE-7, NNE-10 and NNE-20) (Figure 4B). This CpG-rich region in ITGA9 promoter was partially methylated in all NPC samples, whereas only a few CpG sites were partially methylated in the control samples. To investigate the methylation status of WNT7A, sequencing of the clones derived from bisulfite converted DNA was performed at a CpG-rich region in its promoter (Figure 5A). This region contained 84 CpG sites. Four NPC biopsies (NPC-1, NPC-2, NPC-5 and NPC-6) and three control nasopharyngeal epithelia biopsy (NNE-3, NNE-7 and NNE-10) were sequenced (Figure 5B). This CpG-rich region was partially methylated in the four NPC samples. There was virtually no methylation in the two control samples (NNE-3 and NNE-10) while one control sample (NNE-7) showed some methylation at several CpG sites. Taken together these results suggested that particularly ITGA9 could be a candidate TSG in NPC worthy of further analysis.

### Hypermethylation of ITGA9 confirmed in NPC samples by pyrosequencing of the bisulfite converted tissue DNAs

**Figure 6:**
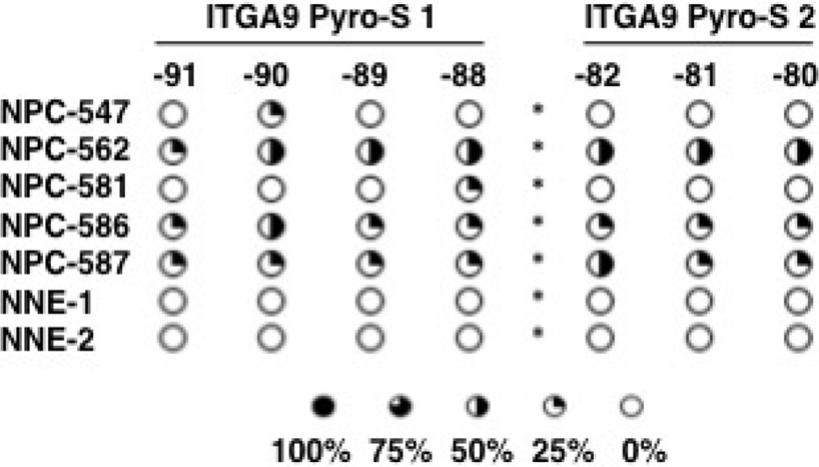
ITGA9 is differentially hypermethylated in NPC clinical samples Methylation status of two subsets of CpG dinucleotides present in one CpG-rich region in ITGA9 gene promoter investigated by sequencing the bisulfite converted DNA using two pyrosequencing assays. Pyro-S1 and pyro-S2 contained four and three CpG sites respectively (see figure 4B). Open and filled circles represent unmethylated and methylated CpG sites, respectively. Circles are filled according to the fraction of sequences methylated at the CpG sites.

Two sub regions with high density CpGs in the ITGA9 control region were analyzed with pyrosequencing of the bisulfite converted tissue DNA. Two pyrosequencing assays (Pyro-S1 and Pyro-S2) targeted two different sets of CpGs that were located in the same region that was subjected to the sequencing of the clones derived from bisulfite converted DNAs as shown (Figure 4A). Pyro-S1 contained four CpGs. Three out of the four CpGs in pyro-S1 and all three CpGs covered by pyro-S2 overlapped with the CpGs used for sequencing the bisulfite converted DNA driven clones. Five biopsies (NPC-547, NPC-562, NPC-581, NPC-586 and NPC-587) and two control nasopharyngeal epithelia biopsies (NNE-1 and NNE-2) were sequenced with this method (Figure 6). The majority of the CpGs in ITGA9 gene promoter were partially methylated in all the NPC samples, while they were unmethylated in the control samples.

### Downregulation of ITGA9 in NPC tissue samples confirmed by Q-PCR

**Figure 7:**
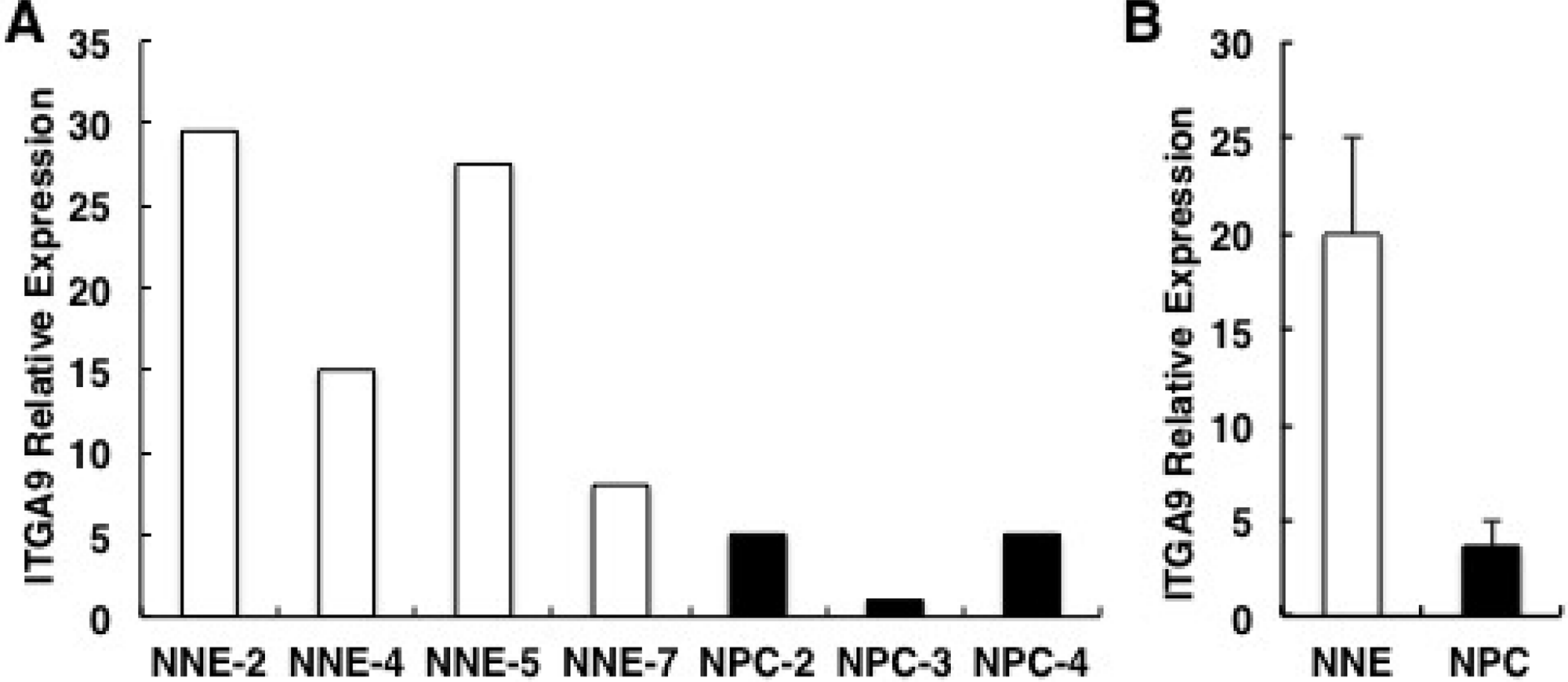
ITGA9 mRNA is downregulated in NPC biopsies **A.** ITGA9 mRNA expression analyzed by Q-PCR in three NPC tissue (T) and four control epithelial tissue samples (N) **B.** The average downregulation of ITGA9 mRNA expression level comparing the three tumors versus four control tissues (from A).

Q-PCR was performed to detect ITGA9 expression in four control epithelial tissues (NNE2, NNE4, NNE5 and NNE7) and three NPC samples (NPC2, NPC3 and NPC4) (Figure 7A). The average expression level of ITGA9 in NPC tumors and in control epithelial tissues was as 4.0 ± 1.3 in NPC vs. 20.0 ± 5.1 in the control samples, respectively (Figure 7B). ITGA9 expression was downregulated in NPC tumor samples as compared to control nasopharyngeal epithelium.

### Screening of methylation in ITGA9- and WNT7A-promoters in NPC biopsy samples from Morocco using MSP

**Figure 8:**
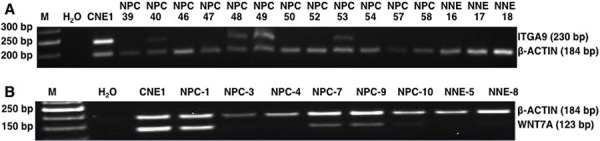
MSP analysis of NPC biopsies and non-cancerous control samples from Morocco Methylation status of ITGA9 **A.** and WNT7A **B.** investigated in an independent cohort of NPC biopsy and control DNA samples by MSP. NPC: tumor; NNE: control nasopharyngeal epithelium.

Methylation specific PCR was used to screen DNA from 36 EBV positive NPC biopsy samples and 18 non-cancerous control samples from Morocco in order to determine the methylation status of ITGA9 and WNT7A. ITGA9 methylation was detected by MSP in 56% (20/36) of NPC DNA samples with 100% specificity (0/18 of non-cancerous control; Figure 8A). WNT7A methylation was detected by MSP in 69% (25/36) of NPC DNA samples with 83% specificity (3 out of 18 in non-cancerous control; Figure 8B). These results suggest that ITGA9 could be a TSG in NPC and promoter hypermethylation could be one mechanism for ITGA9 downregulation in NPC. This might also suggest that ITGA9 could be used as an early detection marker for NPC.

## DISCUSSION

NPC shows several unique features among tumors; its regional very high incidence in Southern China and surrounding countries and its strong association with EBV [31]. The effort has been intense to identify possible susceptibility genes and regional environmental factors, as well as to understand the role of EBV in NPC pathogenesis. Integration of these facts will help in developing prevention, improving diagnosis and treatment [32].

The development of NPC involves the accumulation of multiple genetic changes affecting certain tumor suppressor genes (TSG) or oncogenes thus promoting the clonal evolution of the neoplastic cell, like in many other cancers [33]. TSGs are suggested to play a key role in the transformation of nasopharyngeal epithelial cells into malignant ones [19, 34, 35]. Similarities of patterns in the chromosomal abnormalities have been reported in the NPC from different geographical regions [33]. Chromosome 3p21.3 was the first tumor suppressive region mapped in NPC [36]. This region was shown to contain several TSGs [28, 37–39]. This suggests that inactivation of certain TSGs on chromosome 3 by mutations or epigenetic modifications might play a critical role in NPC [40].

In NPC, a vast number of TSGs are silenced by epigenetic mechanisms, primarily by promoter hypermethylation [41, 42]. It is highly interesting that EBV-infection is associated with extensive DNA methylation in EBV carrying malignancies, also recently demonstrated in EBV-associated gastric cancer [42, 43]. A comprehensive knowledge of hypermethylated and downregulated TSGs is not only important for the development of clinical strategies of NPC prevention and therapy, but hypermethylated TSGs can also be used as diagnostic biomarkers for NPC risk assessment, early detection and prognosis [41]. Many key TSGs for instance p53 and retinoblastoma (Rb) that are frequently reported to be mutated in 50% of all other tumors have been shown to be wild-type in NPC [44, 45]. p16 and Ras association domain family member 1A (RASSF1A) were the first TSGs that were reported to be hypermethylated in NPC [46, 47]. Expression of p53 has also been reported to be deregulated by ubiquitination because of the methylation dependent silencing of UCHL1 - a TSG in NPC [48]. Furthermore hypermethylation of several other TSGs such as retinoic acid receptor β2 (RARβ2), death-associated protein kinase (DAPK), deleted in lung and esophageal cancer 1 (DLEC1) has also been reported in NPC [41, 49–51].

Our established approach was tested here, namely NotI microarray which is a screening method for aberrantly methylated CpG-rich regions in the DNA using methylation sensitive restriction enzymes. It was applied to chromosome 3, which is one of the regions always affected in NPC as well as in many other epithelial cancers [52, 53]. With this screening, ten high scoring candidate genes were identified of which ITGA9 was verified to be methylated and downregulated in NPC tissue samples and NPC cell lines at mRNA and protein levels, which correlated to methylation status of its control regions. Methylation of ITGA9 gene promoter was observed in NPC tumor biopsies despite the challenge of contamination by normal stromal tissue. It suggests that ITGA9 gene potentially could have important functions in tumor tissue organization and its epigenetic inactivation appears to be a major mechanism for its loss of expression in NPC.

The ITGA9 gene is located in the 3p21.3–22.2 segment of chromosome 3 [54, 55] and encodes an integrin subunit, α9. This is a 1035 amino acid polypeptide with a large N-terminal extracellular domain with seven conserved repeats, a transmembrane segment and a short C-terminal cytoplasmic tail. The α9 subunit forms heterodimers only with the β1 chain forming a unique integrin, α9β1, widely expressed in the human airway epithelial cells, and binding to a variety of ligands [56]. The α9β1 integrin receptor plays an integral role in different signal transduction pathways controlling cellular proliferation and differentiation. Integrin α9β1 has been reported to mediate cell migration in glioblastoma [57]. In ITGA9 knockout mice, abnormal proliferation and differentiation of keratinocytes suggest its role in these cellular processes [58]. The role of ITGA9 and its ligands in NPC development deserve further investigation.

Downregulation of ITGA9 has been reported in human papilloma virus associated head and neck squamous cell carcinoma [59], non-small cell lung cancer (NSCLC) [60, 61], leukoplakia, lichen planus, and oral squamous cell carcinoma [62, 63]. Hypermethylation of ITGA9 was found in tumor samples from breast, kidney, cervix, ovary, lung, prostate [30], colorectal cancer [64], hepatocellular carcinoma [65] and premalignant cervical lesions [66]. In another study hypermethylation of ITGA9 at its first intron has been reported to be the major mechanism for its downregulation in breast cancer [67]. Our data is consistent with these findings. Mutations in ITGA9 are a less frequent event while its downregulation is considered to be more important in tumorigenesis [68]. However in contrast to the downregulation of ITGA9 in some cancers it has also been found to be abundantly expressed in small cell lung cancers (SCLC) primary tumors and cell lines where it has been suggested to play a role in metastasis, in the progression to more malignant phenotypes [69] and to poor survival [70]. Similarly integrin α9β1 expression in breast cancer has also been reported to be associated with poor patient survival, increased cell migration and invasion in the breast cancer cell lines [71].

It is interesting to compare results of this study with the work of Law et al [72]. They investigated methylation pattern of chromosome 3 genes in NPC cell lines, and they described five genes (FBLN2, TMEM45A, ZIC4, GPR149 and ETV5) as epigenetically regulated. We were unable to find these genes to be differentially methylated in NPC samples. This could be because we used NPC tissue samples together with NPC cell lines for the initial screening using NMA on chromosome 3.

We have recently evaluated the potential use of ITGA9 methylation as one of the markers in a multiplex methylation specific PCR assay designed for the early diagnosis of NPC patients from different ethnic and geographic backgrounds. The results show that the assay could identify 91% (58/64) NPC patients with 90% specificity (18/20) [73]. Our results strongly suggest that in depth studies should be pursued to understand the functional role of ITGA9 downregulation in the development of NPC.

## MATERIALS AND METHODS

### Cell lines and clinical samples

Human NPC cell lines CNE1 (EBV negative, from the Cancer Center, Sun Yat-Sen University, China) and TWO3 (EBV negative, a gift from Dr. Lin CT, National Taiwan University Hospital) [74] were cultured in IMEM (Gibco) containing 10% fetal calf serum (FCS). NPC cell line C666–1 (EBV positive, from Dr. Huang DR, the Department of Anatomical and Cellular Pathology and Institute of Molecular Oncology, the Chinese University of Hong Kong, Prince of Wales Hospital, Shatin, New Territories, Hong Kong, China) was cultured in IMEM (Gibco) containing 15% FCS. The immortalized nasopharyngeal epithelial cell line NP69 (EBV negative, from Dr. Tsao SW, the University of Hong Kong, China) [75] was cultured in keratinocyte serum-free medium (Invitrogen) supplemented with 25 μg/ml bovine pituitary extract, and 0.2 ng/ml recombinant epidermal growth factor, as suggested by the manufacturer. Cell lines C666–1 and NP69 are authenticated to be of human NPC and NNE origin respectively. C666–1 was also EBV positive. CNE1 and TWO3 are also authenticated to be of human nasopharyngeal epithelial cancer origin, comply with their original profile, but their origin from NPC cannot definitely be verified and they were EBV negative.

Paraffin sections from 20 cases of untreated NPC, and 10 control nasopharyngeal tissues were collected at the Cancer Center, Sun-Yat-Sen University in 2011. For immunostaining, three archival formalin-fixed, paraffin-embedded (FFPE) tissue specimens, which contained both NPC tumor tissue and control epithelial tissue, also had been sampled at the Sun-Yat-Sen University Cancer Center (Guangzhou, China) histopathology bank. This study was approved by the Research Ethics Committee of Sun Yat-Sen University Cancer Center, Guangzhou, China (Reference number: YP-2009175) and the regional ethics committee of the Karolinska Institutet, Stockholm, Sweden (Reference number: 00-302).

NPC DNA samples from 36 EBV positive pathology-verified NPC patients and 18 normal volunteers were obtained from Institut Pasteur du Maroc, Casablanca, Morocco in the year 2011 (ethical approval: No. 00-302, Stockholm, Sweden and 2010-02-15, Casablanca, Morocco) for MSP analysis.

### DNA and RNA extraction

DNA was extracted from NPC cell lines, biopsies and control epithelial tissues, and purified by conventional phenol/chloroform and ethanol extraction. Total RNA was extracted by TRIzol^®^ reagent (Invitrogen, Cat#: 15596-026). All RNA samples were treated with RNAse free DNAse I (Fermentas, Cat#: EN0521) and cDNA was synthesized using MMLV reverse transcriptase with random hexamers according to the standard protocol provided by the manufacturer (Invitrogen, Cat#: AM2043).

### Screening for methylated CpG-rich regions in gene promoter by NotI microarrays

NotI microarrays were constructed as previously described [29, 76, 77]. In the present study, a total 188 putative TSGs were plated in triplicates on glass slides for hybridization screening.

### DNA conversion by bisulfite treatment

DNA samples from NPC patients and controls were processed for bisulfite conversion using EZ DNA Methylation Kit (ZymoResearch, Cat#: D5001) following the manufacturer's recommendations. Five hundred ng of genomic DNA was used as starting material for bisulfite modification.

### 5′-aza-C treatment

NPC cell lines CNE1, TWO3, C666–1 and normal nasopharyngeal epithelium derived cell line NP69 were seeded at 2 × 10^5^ per well in six-well plates and – after 24 hours - cultured with 10 μM 5′-aza-C (Sigma-Aldrich). The medium with the added drug was replaced every 24 hour. After four days treatment, DNA was isolated for measuring the methylation status by MSP, and RNA for evaluating restoration of gene expression by Q-PCR, comparing with medium control.

### Q-PCR assays

The first-strand cDNA was used as template for measuring the expression levels of ITGA9 and WNT7A by mRNA quantitative real time PCR (Q-PCR) with the SYBR Green I chemistry (Power SYBR Green PCR Master Mix, CAT#: 4367659, ABI Inc.). GAPDH was used as internal control. Melting temperature for Q-PCR was 55.5°C, 58°C and 51.5°C for ITGA9, WNT7A and GAPDH respectively. The primer sequences used for Q-PCR are shown in Table 2. The relative expression level was determined as 2-ΔΔCt. Data are presented as the expression level relative to the calibrator control sample.

**Table 2:** Analysis of DNA and RNA: Primers in relation to assays used in the study

	Size	Gene	Amplification Primers (5′ - 3′)	Sequencing Primers (5′ - 3′)	*Modified Sequence/corresponding unmodified sequence analyzed (5′ - 3′)
**Q-PCR**	346 bp	ITGA9-F	CCCCGCTGACTCGTTCTT		
		ITGA9-R	TAGGATGTGGTCGGCTTC		
	487 bp	WNT7A-F	GGGACTATGAACCGGAAAGC		
		WNT7A-R	CGATGCCGTAGCGGATGT		
	67 bp	GAPDH-F	AGCCACATCGCTCAGACAC		
		GAPDH-R	GCCCAATACGACCAAATCC		
**BS-cloning sequencing**	341 bp	ITGA9-F	TTGAGTGGGATTTGAGGATTTGTAT		
		ITGA9-R	ACCTATCTCCCTACCTTTATCTCTCTTTC		
	683 bp	WNT7A-F	TTTAGGTTGAGAAAGAGGTGGTTTA		
		WNT7A-R	AACAAAACAACAAACCAAAACACTA		
**BS-pyrosequencing**		ITGA9-S1-F	AGTGGGATTTGAGGATTTGT	AGGATTTGTATTT TTTGATAAG	TTTTTAGATGA**YG**TTGATGTTGTTGGTT**YG**GAG ATTATATTT**YG**GGAATTATTGGTGTA**YG**AGATTA AGTGATTAGAATATGGAG
		ITGA9-S1-R	biotin-CCCTCCATATTCTAATCACTTAAT		*TTCCCAGATGA**CG**TTGATGCTGCTGGTC**CG**GA GACCACACTT**CG**GGAATCACTGGTGTA**CG**AG ACCAAGTGACTAGAATATGGAG*
		ITGA9-S2-F	ATTGGGTGAAGGAGTAGAGGA	GAAGGAGTAGA GGATG	**YG**TAGGGTTAGAATTTGGAGTTTAAATT**YG**TT TTTTATT**YG**GGTTGTTTTGGATTTTATTGGTTG
		ITGA9-S2-R	biotin-CCCTCCCTATCTCACAACCAATAA		***CG**CAGGGCTAGAACCTGGAGTCTAAACT**CG**T CCTTCACC**CG**GGTTGCTCTGGACTTACTGGTTG*
**MSP**	230 bp	ITGA9-F	GTTGTTGGTTCGGAGATTATATTTC		
		ITGA9-R	AAAACAACCCGAATAAAAAACG		
	123 bp	WNT7A-F	GTAGTTCGGCGTCGTTTTAC		
		WNT7A-R	CGAAACCGTCTATCGATACG		
	184 bp	β-ACTIN F	AAGTTAAGTTTTGTTTTTATTTTTTTT		
		β -ACTIN R	CAATAATCTCCTTCTACATCCTATC		

* The cells with italic text show the unmodified sequences analyzed by bisulfite pyrosequencing.

### Immunostaining

Primary antibodies against ITGA9 (1:100 dilution; Cat#: sc-68864, Santa Cruz Biotechnology, Inc.) and WNT7A (1:250 dilution; Cat#: HPA015719, Atlas Antibodies) were used. Briefly, tissue sections were de-waxed, incubated with hydrogen peroxide for 10 minutes, incubated in retrieval buffer solution for antigen recovery, blocked with normal serum for 10 minutes and incubated with a primary antibody for 60 minutes, followed by detection using a Catalyzed Signal Amplification Kit (DAKO); signals were visualized using diaminobenzidine. Non-immune goat or rabbit serum was substituted for the primary antibody as a negative control.

### Sequencing of clones derived from bisulfite converted DNA

Bisulfite modified DNA was subjected to PCR with primers flanking the targeted methylation-specific PCR regions. The primers used for bisulfite sequencing of ITGA9 and WNT7A were designed using Methprimer [78] online software and are shown in Table 2. PCR products were purified using Invisorb^®^ Spin DNA Extraction Kit (Invitek, Cat#: 1020110200). The purified PCR products were cloned in E.coli TOP10 using CloneJET PCR Cloning Kit (Fermentas, Cat#: K1231). Clones were sequenced using pJET1.2 reverse sequencing primer, 24-mer (Fermentas, Cat#: SO511), a BigDye Terminator v3.1 Cycle Sequencing Kit (Applied Biosystems, Cat#: PN4337035) and 3730 DNA Analyzer (Applied Biosystems). The manufacturers' recommendations were followed during all the steps for bisulfite sequencing.

### Pyrosequencing of bisulfite converted DNA

Two subsets of CpGs within or close to the ITGA9-promoter region sequenced from the clones (above; Figure 4) were used for pyrosequencing after bisulfite-conversion. For pyrosequencing DNAs from same set of NPC tissue and NNE samples were used as in the sequencing of clones. They included five NPC tumors and two control nasopharyngeal epithelial biopsies. The bisulfite pyrosequencing was performed according to the Pyrosequencing Assay Design Software v2.0 (QIAGEN), details of employed sequences are provided in Table 2. Two μl of bisulfite modified genomic DNA (∼20 ng) was used as a template in PCRs performed with the PyroMark PCR kit (QIAGEN, Cat#: 978703) following the manufacturer's recommendations. A melting temperature of 58°C was used for both the assays. PCR amplifications were performed in 50 μl volumes. The entire PCR product, 4 μM of the respective sequencing primer and 2 μl of streptavidin sepharose high-performance beads (GE Healthcare, Cat#: 17-113-01), were used for bisulfite pyrosequencing performed with the PSQ 96 ID system with enzymes and substrates from the PyroMark Gold Q96 reagent kit (QIAGEN, Cat#: 972804). PyroMark Q96 ID software 2.5.8.15 (QIAGEN) was used for data analysis.

### Methylation specific PCR (MSP)

The sequences of PCR primers specific for methylated ITGA9 and WNT7A [79] and the primers for β-ACTIN that served as a quality control to amplify bisulfite-converted DNA without distinguishing between methylated and unmethylated CpGs are given in Table 2. For PCR reaction, 4 μl of bisulfite-modified DNA (40 ng) was added in a final volume of 25 μl of PCR mixture containing 1.8× PCR buffer, 5 mM MgCl_2_, 100 pmol deoxynucleotide triphosphates, primers (100 pmol each per reaction) and one unit of Taq Platinum (Invitrogen). PCR amplifications were performed at 95°C for 3 min, followed by 4 cycles at 94°C for 1 min, 60°C for 30 s, and 72°C for 45 s, which was then followed by 28 amplification cycles at 94°C for 1 min, 56°C for 1 min, and 72°C for 45 s. The final elongation step was performed at 72°C for 4 min. MSP products were analyzed by 2% agarose gel electrophoresis stained with ethidium bromide.
